# Greenhouse gas emissions and carbon footprint of collard greens, spinach and chicory production systems in Southeast of Brazil

**DOI:** 10.3389/fpls.2022.1015307

**Published:** 2022-11-02

**Authors:** Breno de Jesus Pereira, Arthur Bernardes Cecílio Filho, Newton La Scala, Eduardo Barretto de Figueiredo

**Affiliations:** ^1^ College of Agricultural and Veterinarian Sciences, São Paulo State University - Sao Paulo State University (UNESP), São Paulo, Brazil; ^2^ Department of Rural Development, Federal University of São Carlos (UFSCar), São Paulo, Brazil

**Keywords:** vegetables, global warming potential, intercropping, direct and indirect emissions, carbon footprint

## Abstract

Food production in sustainable agricultural systems is one of the main challenges of modern agriculture. Vegetable intercropping may be a strategy to mitigate greenhouse gas (GHG) emissions, replacing monoculture systems. The objective is to identify the main emissions sources and to estimate GHG emissions of intercropping and monoculture production of collard greens, New Zealand spinach and chicory. Four scenarios were evaluated: ICS – intercropping collard greens and spinach; MCS – monoculture collard greens and spinach; ICC – intercropping collard greens and chicory; MCC - monoculture collard greens and chicory. The boundaries’ reach from “cradle-to-gate” and the calculation of GHG emissions were performed using IPCC methodology and specific factors (Tier 2). The total GHG emitted was standardized as CO_2_ equivalent (CO_2_eq). The GHG emissions in ICS and ICC scenarios were approximately 31% lower than in MCS and MCC scenarios. Carbon footprint in ICS (0.030 kg CO_2_eq kg^-1^ vegetables year^-1^) and ICC (0.033 kg CO_2_eq kg^-1^ vegetables year^-1^) scenarios were also lower than in MCS (0.082 kg CO_2_eq kg^-1^ vegetables year^-1^) and MCC (0.071 kg CO_2_eq kg^-1^ vegetables year^-1^) scenarios. Fertilizers, fuel (diesel) and irrigation were the main contributing sources for total GHG emitted and carbon footprint in all evaluated scenarios. The results suggest that intercropping systems may reduce GHG emissions associated with the production of vegetables evaluated as compared with monoculture.

## Introduction

In the last few decades, the production of leafy vegetables has been rising and standing out in a world agricultural context (FAO, 2019), consequence of the increasing demand for food and changing in feeding habits (Vico et al., 2020). The accelerated increase of population and the need to produce food for eight billion people lead to a huge environmental impact, mainly on climate change/global warming, since conventional agricultural system (monoculture) is characterized by intense exploration of natural resources (soil and water) and large use of inputs, materials and fuel (fertilizers, pesticides, diesel, plastic etc.), increasing direct and indirect greenhouse gas emissions (GHG) (Notarnicola et al., 2017).

Improving food production systems aiming at making them more sustainable is the main challenge of agriculture in the current century (Foteinis and Chatzisymeon, 2016). Compared with conventional systems, sustainable agricultural systems are characterized by reduced use of chemical fertilizers, pesticides, fuel and lower impact on natural resources (soil and water) (Jeswani et al., 2018; Seo et al., 2019) and GHG emissions (Pereira et al., 2021). These changes applied in the vegetable production sector may directly contribute to achieve some of the main goals proposed by United Nations (UN) aiming at the sustainable development, such as development of sustainable agriculture, responsible consumption and production, and climate action (UN, 2015).

Vegetable production, performed mainly in monoculture systems, contributes directly climate change/global warming due to greenhouse gas (GHG) emissions generated by intensive soil tillage and use of fertilizers and fuels (Martin-Gorriz et al., 2020; Pereira et al., 2021). The challenges brought by climate change/global warming will require strategies of adaptation to meet consumers’ demands and to ensure high standards for food safety (Bisbis et al., 2018). One alternative to food production in monoculture is the intercropping systems of vegetables because, in addition to agroeconomic viability (Cecílio Filho et al., 2017; Carlos et al., 2021), this system has a potential to mitigate GHG emissions (Pereira et al., 2021).

The main agronomic advantage of intercropping is better use of agricultural area since two species are simultaneously cultivated in the same area, increasing diversity of species in the system and reducing the use of inputs, materials and fuel (Nascimento et al., 2018). This system’s agronomic variability depends on temporal and/or spatial complementarity of the species cultivated, as demonstrated in cultivation of collard greens and New Zealand spinach by Cecílio Filho et al. (2017), and in cultivation of collard greens and chicory by Carlos et al. (2021). Therefore, vegetable intercropping is a viable technology to meet the rising demands for food production and reduce the impact on climate change/global warming (Pereira et al., 2021).

Studies identifying the main sources of GHG emissions and the impact of vegetable production systems on climate change/global warming have been published for many countries in the last few years (Clavreul et al., 2017; Ntinas et al., 2017; Pishgar-Komleh et al., 2017; Seo et al., 2017; Tasca et al., 2017; Zarei et al., 2019; Lo-Iacono-Ferreira et al., 2020). In Brazil, the only study found in the literature evaluating the impacts of vegetable cropping systems was published by Pereira et al. (2021). The authors demonstrated the potential of intercropping of vegetables to mitigate GHG as compared to monocultures, cultivating vegetables such as tomato, cucumber and lettuce, in greenhouse. However, no publications were found about the potential of mitigation of GHG emissions in the intercropping of other vegetables, such as collard greens (*Brassica oleracea* var. *acephala*), New Zealand spinach (*Tetragonia expansa*) and chicory (*Cichorium intybus*), which are addressed in this study.

Collard greens are leafy vegetables cultivated at about 71.279 agricultural farms in Brazil, which produce approximately 161.986 tons per year (IBGE, 2017). Therefore, evaluating the impact on climate change/global warming to produce this vegetable, intercropped with New Zealand spinach or chicory, and compared to their respective monocultures, will be important to suggest agricultural practices with lower GHG emissions and higher yield. As demonstrated by Pereira et al. (2021), intercropping of vegetables may reduce GHG emissions by 35% in comparison to monocultures. In addition, the authors demonstrated that carbon footprint may be five times lower in the intercropping when compared to monocultures.

In this context, our study aimed to calculate GHG emissions and carbon footprint in two production systems (intercropping and monoculture) of collard greens, New Zealand spinach and chicory, arranged in four scenarios: 1) intercropping of collardgreens and New Zealand spinach; 2) monocultures of collard greens and New Zealand spinach; 3) intercropping of collard greens and chicory; 4) monocultures of collard greens and chicory; and to identify the main sources of GHG emissions, suggesting to mitigation practices. Our hypothesis is that intercropping systems to produce collard greens, New Zealand spinach and chicory are responsible to lower greenhouse gas emissions and lower carbon footprint when compared to monoculture systems.

## Material and methods

### Description of production scenarios

**Figure 1:**
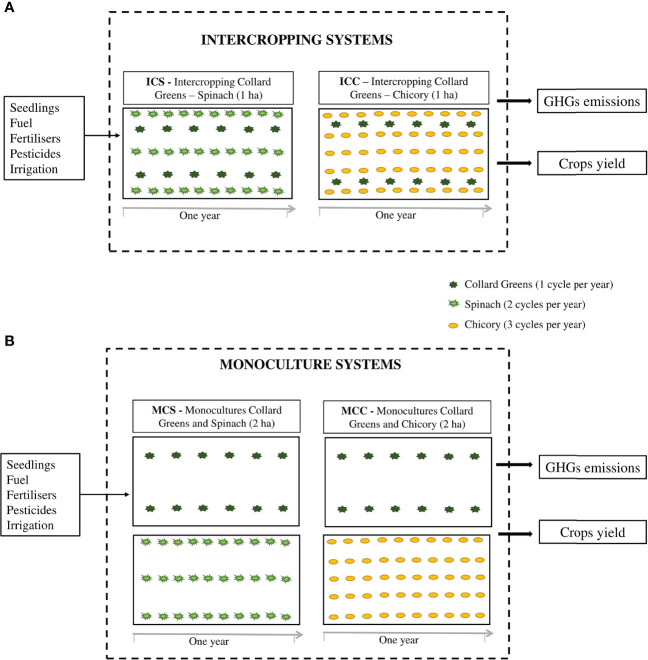
Vegetable production scenarios evaluated: **(A)** intercropping: ICS – intercropping of collard greens and spinach (area of 1 ha); ICC – intercropping of collard greens and chicory (area 1 ha); **(B)** monocultures: MCS – monocultures of collard greens and spinach (2 ha); MCC – monocultures of collard greens and chicory (2 ha); and their respective sources of GHG emissions during one agricultural year of production.

GHG emissions and carbon footprint were evaluated in four scenarios of production of collard greens and New Zealand spinach (Cecílio Filho et al., 2017) and collard greens and chicory (Carlos et al., 2021), in Jaboticabal city, São Paulo state, Brazil. The scenarios were defined as follows: 1) ICS – intercropping of collard greens and New Zealand spinach; 2) MCS – monocultures of collard greens and New Zealand spinach; 3) ICC – intercropping of collard greens and chicory; 4) MCC – monocultures of collard greens and chicory (**Figure 1**).

ICS and MCS scenarios consisted of collard greens and New Zealand spinach cultivation in intercropping (ICS) and monocultures (MCS), in open field, during one agricultural year, considering one cycle of cultivation for collard greens (cycle of 12 months) and two cycles for New Zealand spinach (5 months each cycle), with three harvests per cycle of spinach (**Figure 1**) (Cecílio Filho et al., 2017). According to the authors, for ICS, collard greens cv. ‘Top Bunch’ and New Zealand spinach cv. ‘New Zealand’ were planted simultaneously in the same area, in beds with two rows of collard greens (double rows – spaced by 0.50 × 0.50 m) and three rows of New Zealand spinach (spaced by 0.40 × 0.30 m). For MCS, using the same species and spacing of planting, the plants were cultivated in different areas (**Figure 1**).

For ICC and MCC scenarios, one year of cultivation of collard greens and chicory in intercropping (ICC) and monoculture (MCC) systems, in open field, were considered, one cycle of collard greens (cycle of 12 months) and three cycles of chicory (4 months each cycle), with two harvests per cycle of chicory (**Figure 1**) (Carlos et al., 2021). According to the authors, for ICC, collard greens cv. ‘HS-20’ and chicory cv. ‘Pão de Açúcar’ were cultivated in the same bed, with two rows of collard greens (double rows – spaced by 0.70 × 0.40 m) and five rows of chicory (spaced by 0.25 × 0.20 m). For MCC, using the same species and spacing of planting, the plants were cultivated in different areas (**Figure 1**).

### Functional units

Aiming to compare inputs and outputs for each scenario, threes functional units were defined to be used in this study: one kilogram of vegetables (kg vegetables year^-1^), one kilocalorie of vegetables (kcal vegetables year^-1^), produced during one year of cultivation, and one hectare of cultivation (ha vegetables year^-1^). GHG emissions were calculated using the methodology of Intergovernmental Panel on Climate Change (IPCC, 2006) and other specifics factors (Tier 2). All factors used can be found in **Supplementary material – Table 1**. Total GHG emissions were calculated in CO_2_ equivalent (CO_2_eq), considering Global Warming Potential equal to 1 for carbon dioxide (CO_2_), equal to 28 for methane (CH_4_) and equal to 268 for nitrous oxide (N_2_O) over a given period of 100 year (IPCC et al., 2013).

### Boundaries established for the study

The boundaries established for this study include agricultural phase of production of collard greens, New Zealand spinach and chicory and transportation of seedlings and fertilizers (cradle-to-gate analyses) for each scenario (**Figure 1**). Sources of GHG emissions in the boundaries established were classified into five categories: seedling production (polypropylene trays and greenhouse structure); fuel (diesel used in the transportation of seedlings and fertilizers, and in the operations using tractor); fertilizers (NPK, limestone and organic fertilizers); pesticides (insecticides and fungicides); irrigation (PVC tubes, sprinklers, and electricity) (**Figure 1**).

In ICS and ICC scenarios there is an overlap of cultivation area, that is, secondary crops (New Zealand spinach and chicory) are cultivated between the main crop (collard greens) rows, using the same spacing of planting as the monocultures. In MCS and MCC scenarios the crops are cultivated individually in two different areas (one area for each species present in the intercropping) because this is the principle of the monoculture. However, both systems (intercropping and monoculture) have the same number of plants because the same spacing of planting was used, that is, one hectare of intercropping has the same number of plants for each species as two hectares of monoculture (**Figure 1**).

Thus, aiming to portray the real condition of each cultivation system, the estimates of GHG emissions in the production scenarios were made by comparing one hectare of intercropping with two hectares of monoculture, being one hectare of monoculture for each species present in the intercropping (**Figure 1**). Carbon footprint to produce one kilogram of vegetables was determined by dividing total GHG emissions in each production scenario (kg CO_2_eq ha^-1^ year^-1^) by the total yield for each crop (kg vegetables ha^-1^ year^-1^), adding the partials to obtain the total in each scenario (**Figure 1** and **Table 2**). To calculate carbon footprint in kilocalories (kcal), total crop yield was converted into kcal using values of caloric composition in 100 g of fresh vegetables, equal to 27 kcal 100 g^-1^ collard greens, 16 kcal 100 g^-1^ New Zealand spinach and 18 kcal 100 g^-1^ chicory (TACO, 2011). After the total of kcal was calculated, carbon footprint to produce one kcal of vegetables was determined by dividing total GHG emissions in each production scenario (kg CO_2_eq ha^-1^ year^-1^) by the total energy yield of each crop (kcal vegetables ha^-1^ year^-1^), adding the partials to obtain the total in each scenario (**Figure 1** and **Table 2**). In this study, the CO_2_ absorbed by plants was disregarded.

### NPK fertilizers, liming and organic fertilizers

The amounts of N used in fertilization at planting, in ICS and ICC scenarios, were defined by establishing an average value of the recommendation for collard greens, and for side dress, by adopting an average value of the recommendation for each species (Cecílio Filho et al., 2017; Pereira et al., 2021). In MCS and MCC scenarios an average value of the fertilization recommendation for each species at planting and side dress was adopted (Trani et al., 2018). Total values of the amount of N used (kg N ha^-1^ ano^-1^) are shown in **Table 1**.

**Table 1:** Amount of inputs and materials used for one year of production of collard greens (1 cycle per year), New Zealand spinach (2 cycles per year) and chicory (3 cycles per year) in intercropping and monoculture scenarios.

Source	Unit	ICS ^a^	MCS ^b^	ICC ^c^	MCC ^d^
N fertiliser	kg ha^-1^ year^-1^	350.0	410.0	410.0	500.0
P fertiliser (P_2_O_2_)	kg ha^-1^ year^-1^	420.0	600.0	600.0	780.0
K fertiliser (K_2_O)	kg ha^-1^ year^-1^	280.0	400.0	310.0	490.0
Limestone	kg ha^-1^ year^-1^	500.0	1,000.0	500.0	1,000.0
Manure	t ha^-1^ year^-1^	40.0	80.0	40.0	80.0
Fungicides (i.a ^e^)	kg ha^-1^ year^-1^	0.19	0.29	0.29	0.38
Insecticides (i.a ^e^)	L ha^-1^ year^-1^	1.27	1.91	1.91	2.54
Diesel	L ha^-1^ year^-1^	213.5	411.5	259.5	545.5
Electricity	kwh ha^-1^ year^-1^	1,093.95	2,187.9	1,093.95	2,187.9
Irrigation pipes	kg ha^-1^ year^-1^	159.20	318.40	159.20	318.40
Irrigation sprinkler	kg ha^-1^ year^-1^	4.17	8.33	4.17	8.33
Seedling trays	kg ha^-1^ year^-1^	135.74	135.74	264.0	264.0
Iron ^f^	kg ha^-1^ year^-1^	87.50	87.50	87.50	87.50
Film Plastic ^f^	kg ha^-1^ year^-1^	53.33	53.33	53.33	53.33

a ICS – intercropping collard greens - spinach.

b MCS – monoculture collard greens and spinach.

c ICC – intercropping collard greens - chicory.

d MCC – monoculture collard greens and chicory.

e Active ingredient.

f Seedling greenhouse.

Total of P fertilizer (superphosphate 17 – 18% P_2_O_5_), K fertilizer (potassium chloride 58 – 60% K_2_O), limestone and organic fertilization (manure) used in each scenario were based on the recommendations of Trani et al. (2018) and are described in **Table 1**. Total amounts of limestone for each scenario were divided by three years, considering this the time needed to perform a new application (**Table 1**). Indirect emissions attributed to the manufacturing process of NPK and limestone fertilizers were estimated by factors used in the EBAMM and GREET models, adapted by Macedo et al. (2008), and the IPCC (2006) factor was used to calculate direct emissions associated with the limestone application. The average N content of the manure was 1.7%, and the emission factor used in the calculations of the direct emission was according to Lessa et al. (2014).

### Pesticides

To control pests and diseases, the use of insecticide (Akito – 10% of active ingredient Beta-Cypermethrin), insecticide/acaricide (Oberon – 2% of active ingredient Spiromesiphen) and fungicide/bactericide (Kasumin – 2% of active ingredient Kasugamycin) was considered in all evaluated scenarios in this study. The amount was determined according to the recommendation for each crop (**Table 1**). The use of herbicides was not considered in any of the evaluated scenarios. The factors adapted by Macedo et al. (2008) and by Do Carmo et al. (2016) were used to calculate the indirect emission associated with the manufacturing of insecticides and fungicide, respectively.

### Irrigation – indirect emission

In all evaluated scenarios, the use of sprinkler irrigation system using 75-mm-diameter PVC tubes in the lateral lines and 100-mm-diameter PVC tubes in the main line of the system was considered. The material weight was based on the manufacturer’s information, calculated from the weight of a 6-m-long pipe. The use of 50 sprinklers (12 m x 18 m spacing), weighing 250 g each and mostly manufactured using low-density polyethylene – LDPE, was designed. Lifespans of five years for PVC pipes and three years for sprinklers were considered. Emission factors used in calculating the emissions associated with the manufacture of PVC pipes and sprinklers were according to Posen et al. (2017).

For system operation, the use of a DANCOR cast iron pump (10 hp/7293 Watts), sufficient to irrigate 1 hectare using the adopted irrigation system, was designed. Total electricity consumption was determined by assuming a 30-min daily watering, for a period of 10 months, resulting in 150 hours per year in all scenarios (**Table 1**), using the following equation:


Consumption (kWh) = (Pp × h /1000) × tc

where *Pp* pump power (Watts); *h* hours of operation per month; *tc* time of the crop cycle (in months).

To calculate CO_2_eq emissions associated with energy consumption in irrigation, the average value of emission factors for electricity generation in the Brazilian National Interconnected System according to the Ministry of Science, Technology and Innovations (MCTI, 2020) was used.

### Seedling production

For seedling production, the use of a 624-m^2^ greenhouse, structured with galvanized iron and covered with transparent polyethylene film, 15-mm thick with additives against ultraviolet rays, was considered. Lifespans of 40 years for the iron used in the structure and 3 years for the plastic film were considered. The amount of iron and plastic film used are shown in **Table 1**. However, the emissions referring to the time of use for seedling production, corresponding to two months per year, in all evaluated scenarios were calculated. For sowing of vegetable seedlings, we considered the use of plastic trays with 200 cells of 0.018 dm^3^ each. The trays are manufactured with low-density polyethylene, weighing 1.100 g each. The number of trays used for seedling production was calculated based on the planting spacing of the crops, considering the total of plants in one hectare (**Table 1**). The adopted lifespan of the trays was 5 years.

The GHG emissions to manufacture the iron and the plastic film were calculated according to IPCC (2006) and Cheng et al. (2011), respectively. Emissions associated with tray manufacturing were calculated using a factor according to Posen et al. (2017).

### Diesel – direct and indirect emission

The total diesel consumed in the evaluated scenarios includes diesel consumed in the transportation of seedlings and fertilizers over a distance of 50 km to the cultivation area, transported by a Mercedes Artego semi-heavy truck. In the area of vegetable cultivation, we considered diesel used for ploughing, harrowing, construction of beds, limestone application, cattle manure application and harvest transportation from the field to the shed (established distance of 1 km) (**Tables 1** and **Supplementary Material – Table 2**), using a MF 275 tractor (77 hp). In MCS and MCC scenarios, the operations of ploughing, harrowing and construction of beds are performed at each new cycle in the cultivation areas of spinach and chicory. However, in ICS and ICC scenarios those operations are performed only one time (before the first cultivation), because from the second cycle of spinach or chicory, the collard greens are already growing in the area, making it impossible to perform those operations; therefore, no-till planting of spinach and chicory must be carried out (**Supplementary Material – Table 2**).

Direct CO_2_eq emissions associated with fuel combustion were calculated using the emission factor established by the São Paulo State Environmental Company (CETESB, 2018). For indirect emissions, associated with diesel extraction and production, the factor according to Macedo et al. (2008) was used.

### Variations in soil carbon stock

Changes in soil carbon stock were estimated based on IPCC (2006) factors for a 20-year period. Land use change (F_LU_), soil management (F_MG_) and crop residue deposition (F_I_) factors were defined according to the specific climate, classified as tropical humid, in the São Paulo state (CEPAGRI, 2006) and considering a high soil management intensity, with values of F_LU_ = 0.83, F_MG_ = 1.00 and F_I_ = 0.92. The reference carbon stock value (C_ref_) used was 38 t C ha^-1^, i.e., the IPCC standard value for clay soils (dark red Oxisol), considering a soil depth of 0–30 cm. Thus, the estimates were made using the following equation:


$$\Delta C_{soil}\;=\left(C_{ref}\times \;F_{LU\;}\times F_{I}\times F_{MG}\right)-C_{ref}\;\;\;\;\;\;$$

where Δ*C_soil_* change in soil carbon stock over 20 years (t C ha^-1^); *C_ref_* reference carbon stock for Oxisols (t C ha^-1^); *F_LU_* factor associated with land use change (dimensionless); *F_1_* factor associated with crop residue deposition (dimensionless); *F_MG_* factor related to the adopted soil management practices (dimensionless).

After determining the total soil carbon accumulation/loss, the value found was converted from carbon (C) into carbon dioxide (CO_2_) by multiplying it by the ratio of 44/12, i.e., 1 t of C corresponds to 3.67 t of CO_2_.

## Results

### GHG emissions and soil carbon

**Figure 2:**
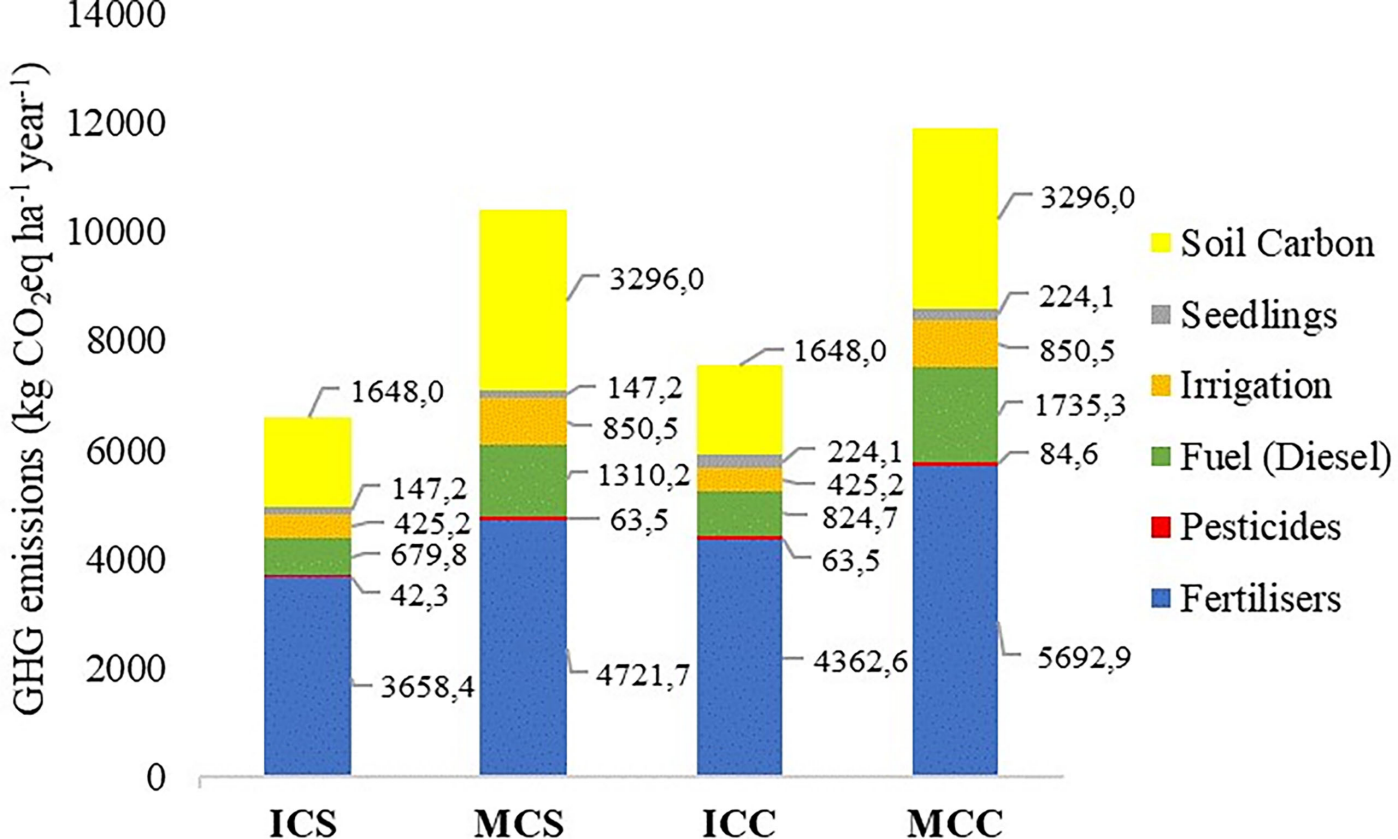
Total GHG emissions (kg CO_2_eq ha^-1^ year^-1^), associated with changes in soil carbon stock and GHG emission sources defined according to the boundaries adopted in the vegetable production scenarios evaluated: ICS – intercropping of collard greens and New Zealand spinach; MCS – monocultures of collard greens and New Zealand spinach; ICC – intercropping of collard greens and chicory; MCC – monocultures of collard greens and chicory.

Total of direct and indirect GHG emissions associated with collard greens and spinach production in ICS scenario were 4,953 kg CO_2_eq ha^-1^ year^-1^ while in MCS were 7,093 kg CO_2_eq ha^-1^ year^-1^. In ICC scenario, total emissions reached 5,900 kg CO_2_eq ha^-1^ year^-1^ while in MCC scenario they reached 8,587 kg CO_2_eq ha^-1^ year^-1^ (**Figure 2**). These results show a reduction of about 31% in GHG emissions when the vegetables evaluated are cultivated in intercropping as compared to monocultures. Such reductions in GHG emissions in ICS and ICC scenarios are mainly related to decrease in fertilizer use, as the species present in intercropping system have a synergy as to fertilizers uptake applied at planting; in fuel (diesel) consumption, as the operations for soil tillage are performed in only one area of cultivation; and in the material cultivation use such as irrigation equipment and electricity consumption, as ICS and ICC scenarios require smaller irrigation system, needed to cover half of the cultivation area when compared to that required in MCS and MCC scenarios, demonstrating a great competitive advantage in reducing GHG emissions in intercropping systems.

When adding the estimates of changes in soil carbon stock due to land use change and soil management, the ICS and ICC scenarios (1,648.0 kg CO_2_ ha^-1^ year^-1^) result in lower carbon losses compared with MCS and MCC scenarios (3,296.0 kg CO_2_eq ha^-1^ year^-1^) (**Figure 2**). The 50% reduction in losses is related to the use of 50% of the cultivated area, since, in intercropping, the two crops grow together in the same area (1 ha), while in monocultures two cultivation areas are needed (2 ha). Thus, ICS and ICC scenarios may be options for production systems with a potential to mitigate GHG emission, as in addition to reducing CO_2_ emissions associated with inputs, there is also a reduction in CO_2_ emissions from losses in soil carbon stock, since it is possible to optimize the production in the same area by using intercropping systems.

In all analyzed scenarios, fertilizer use was the main responsible for GHG emissions, representing about 74% of total emissions in each intercropping scenario (ICS: 3,658.4 kg CO_2_eq ha^-1^ year^-1^ and ICC: 4,362.6 kg CO_2_eq ha^-1^ year^-1^), and about 66% of total emissions in each monoculture scenario (MCS: 4,721.7 kg CO_2_eq ha^-1^ year^-1^ and MCC: 5,692.9 kg CO_2_eq ha^-1^ year^-1^). Among the fertilizers used, nitrogen fertilizer was the major contributor, mainly due to direct (from 20 to 25% of total in the evaluated scenarios) and indirect (from 23 to 28% of total in the evaluated scenarios) emissions associated with this input. In addition to fertilizers, fuel (diesel) accounted for about 14% of total emissions in ICS and ICC scenarios, and for about 18 and 20% in MCS and MCC scenarios, respectively (**Figure 2**).

**Figure 3:**
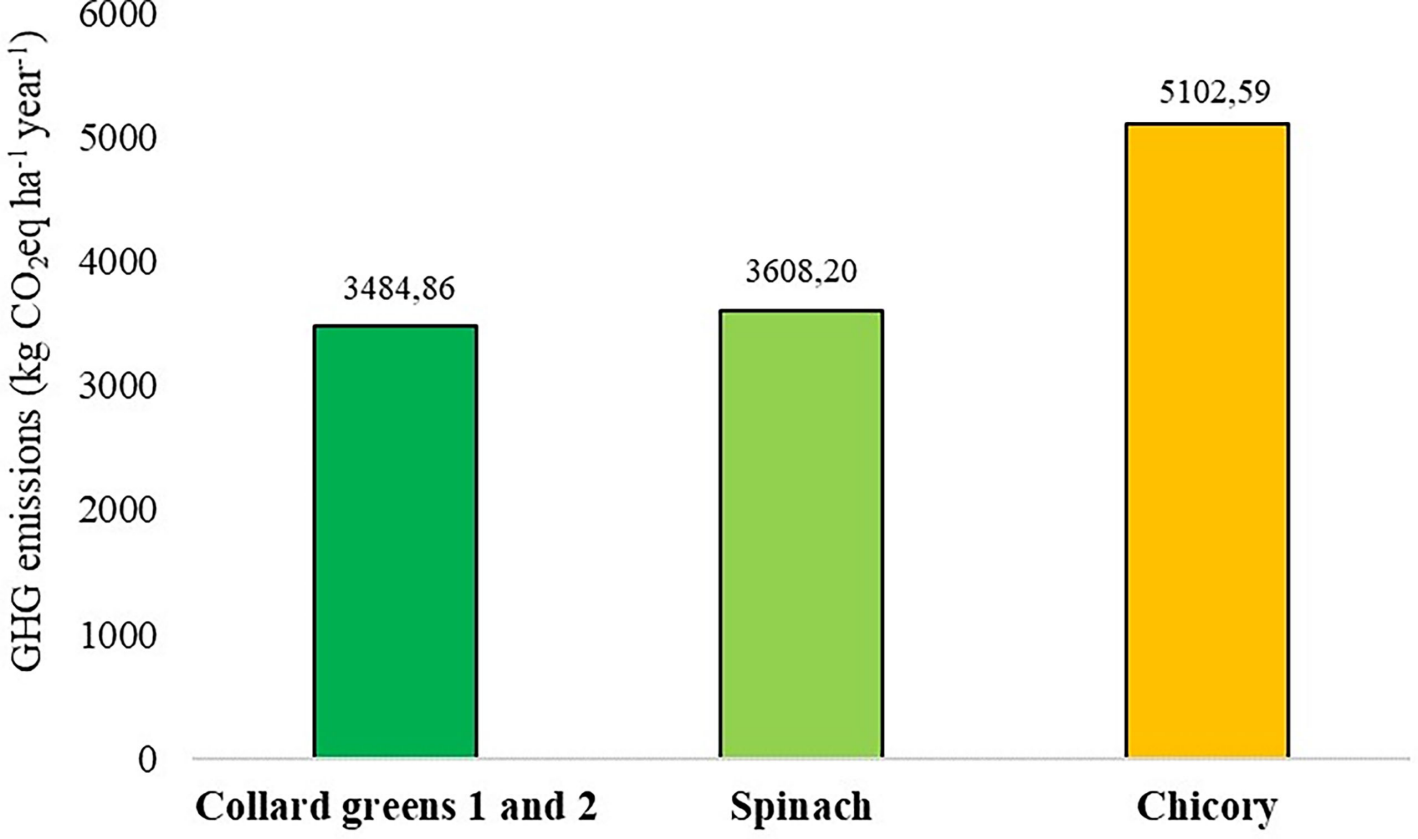
Total GHG emissions (kg CO_2_eq ha^-1^ year^-1^) for individual production of each species in monoculture scenarios: MCS – monocultures of collard greens 1 and New Zealand spinach and MCC – monocultures of collard greens 2 and chicory.

Analyzing the emissions associated with each individual species, in MCS and MCC scenarios, it is possible to observe that chicory production emits 5,102.59 kg CO_2_eq ha^-1^ year^-1^, while the production of New Zealand spinach and collard greens emits 3,608.20 kg CO_2_eq ha^-1^ year^-1^ and 3,484.86 kg CO_2_eq ha^-1^ year^-1^, respectively (**Figure 3**). The highest emissions in chicory cultivation are related to the greater number of crops established during the year, making it possible to carry out three cycles of chicory, two of New Zealand spinach and one of collard greens. In addition, the use of fertilizers, diesel in the operations and transport, pesticides and electricity for chicory cultivation is greater, increasing the emissions associated with these sources.

### Carbon footprint

**Figure 4:**
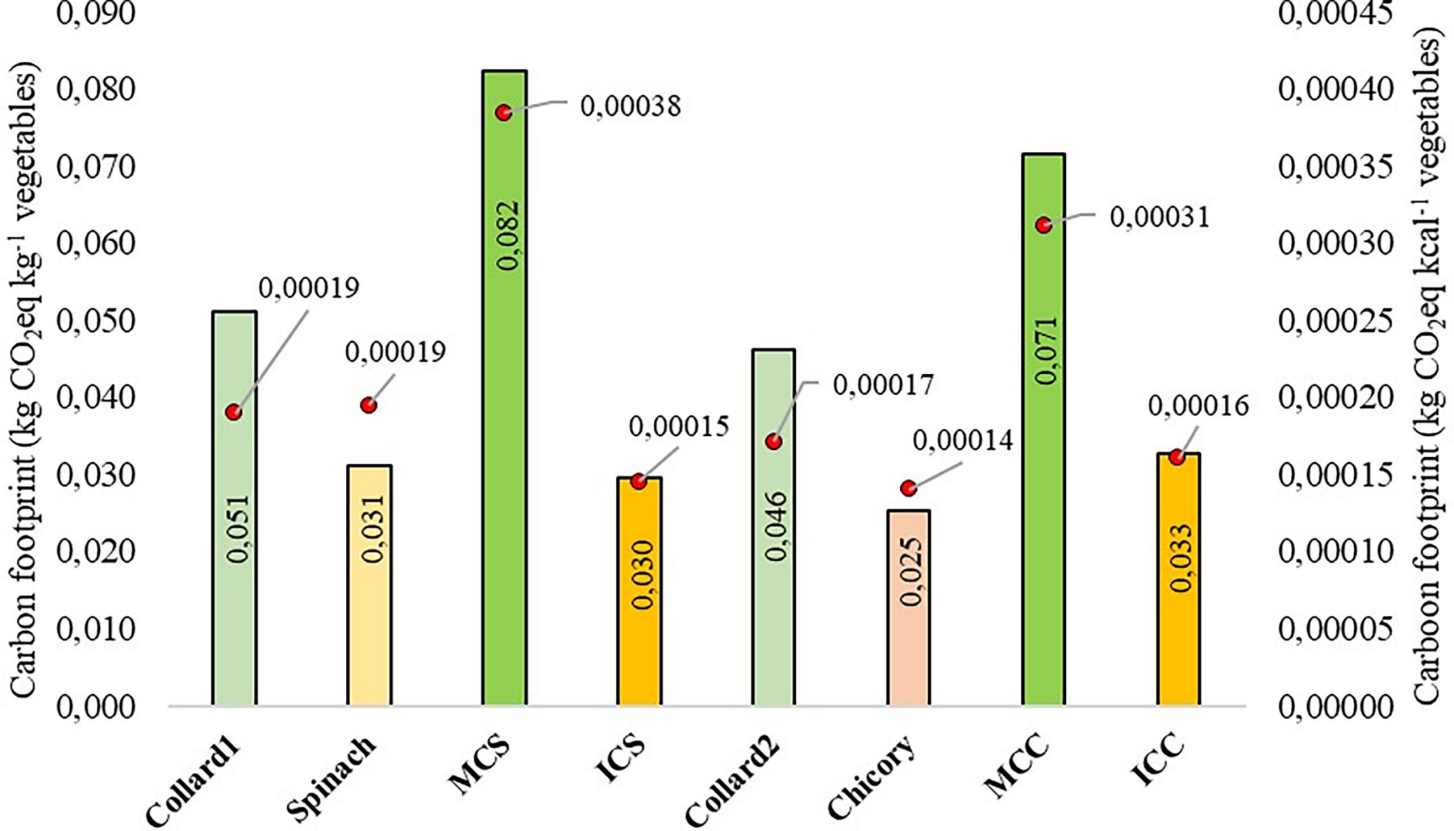
Carbon footprint (Colored bars = kg CO_2_eq kg^-1^ vegetable year^-1^; Red points = kg CO_2_eq kcal^-1^ vegetables year^-1^) for each vegetable species produced and for each system in the evaluated production scenarios: ICS – intercropping of collard greens and New Zealand spinach; MCS – monocultures of collard greens and New Zealand spinach; ICC – intercropping of collard greens and chicory; MCC – monocultures of collard greens and chicory.

Concerning carbon footprint to produce one kilogram of vegetables, in MCS scenario it was 0.082 kg CO_2_eq kg^-1^ vegetables year^-1^ while in ICS scenario it was 0.030 kg CO_2_eq kg^-1^ vegetables year^-1^, which represents a 64% reduction of carbon footprint in ICS scenario when compared with the MCS (**Figure 4**). Carbon footprint was equal to 0.071 kg CO_2_eq kg^-1^ vegetables year^-1^ in MCC scenario and equal to 0.033 kg CO_2_eq kg^-1^ vegetables year^-1^ in ICC scenario, representing approximately 54% of reduction (**Figure 4**). When analyzing the carbon footprint values in kilograms of CO_2_eq per kilocalories of produced vegetables, we observed that the reductions from the MCS scenario to ICS and from the MCC to ICC were 61 and 48%, respectively (**Figure 4**). The reductions of carbon footprint in intercropping scenarios (ICS and ICC) compared to monoculture scenarios (MCS and MCC) is mainly related to the reduction in GHG emissions when vegetables are cultivated in intercropping, since this system has better efficiency in land use and requires less use of fertilizers, fuels and electricity consumption with the irrigation system (**Figure 2**).

In MCS and MCC scenarios it is possible to analyze the carbon footprint for individual crops. In the MCS, the carbon footprint of collard greens was 0.051 kg CO_2_eq kg^-1^ collard greens year^-1^, and for spinach it was 0.031 kg CO_2_eq kg^-1^ spinach year^-1^. However, when analyzing the carbon footprint per kilocalorie of energy produced, it was observed that the values were the same for both species, that is, 0.00019 kg CO_2_eq kcal^-1^ spinach year^-1^ and 0.00019 kg CO_2_eq kcal^-1^ collard greens year^-1^ (**Figure 4**). These results were due to the yield and energy capacity of these species, since despite producing less fresh mass, collard greens (27 kcal 100 g^-1^) are more energetic than spinach (16 kcal 100 g^-1^) (TACO, 2011). In MCC scenario, the values were 0.046 kg CO_2_eq kg^-1^ collard greens year^-1^ (or 0.00017 kg CO_2_eq kcal^-1^ collard greens year^-1^) for collard greens monoculture and 0.025 kg CO_2_eq kg^-1^ chicory year^-1^ (or 0.00014 kg CO_2_eq kcal^-1^ chicory year^-1^) for chicory monoculture (**Figure 4**). The difference in the carbon footprint in collard greens monocultures 1 and 2 are associated with the different yields (**Table 2**), since GHG emission for collard greens is the same in both monoculture scenarios (MCS and MCC) (**Figure 3**). Among the evaluated vegetables, chicory was the one which showed lower carbon footprint (**Figure 4**), but had the highest GHG emission (**Figure 3**). Such result is related the number of cultivation cycles of this vegetable in one year (3 cycles), that is, despite the highest total GHG emission, there is also a higher yield for this vegetable during one year (**Table 2**). Therefore, the higher the crop yield, the smaller carbon footprint per kilogram (Pishgar-Komleh et al., 2017).

**Table 2:** Vegetable yield (t fresh mass ha^-1^ year^-1^ and kcal ha^-1^ year^-1^) of each species (collard greens – one cycle per year; New Zealand spinach – two cycles per year; chicory – three cycles per year) within the cropping systems evaluated and total yield of each cropping system.

Crop systems	Fresh yield	Energy yield
ICS		
Collard greens + New Zealand Spinach	(67.09 ^a^ + 100.16 ^b^) = 167.25	(18,114,300^a^ + 16,026,240^b^) = 34,140.5
MCS		
Collard greens	68.06	18,376,200
New Zealand Spinach	115.78	18,524,160
ICC		
Collard greens + Chicory	(47.87 ^c^ + 132.24 ^c^) = 180.07	(12,924,900^d^ + 23,796,720^d^) = 36,721.6
MCC		
Collard greens	75.39	20,353,680
Chicory	201.99	36,358,200

a Collard greens yield (Cecílio Filho et al., 2017).

b New Zealand Spinach yield (Cecílio Filho et al., 2017).

c Collard greens yield (Carlos et al., 2021).

d Chicory yield (Carlos et al., 2021).

## Discussion

Studies assessing GHG emissions and carbon footprint in the production of collard greens, New Zealand spinach and chicory were not found in literature. However, when assessing the impact of GHG emissions in vegetable production systems in intercropping and monoculture in Brazil, Pereira et al. (2021) found that the intercropping may reduce GHG emissions by 35% in comparison to monoculture production. As in the present study, the authors also found that the decrease in fertilizer use promoted by intercropping was one of the main responsible for reducing GHG emission. It is important to highlight that in the cited study, the authors evaluated the production systems with different vegetable species (cucumber, tomato and lettuce) from those evaluated in this study; however, the results obtained corroborate those of the present study about intercropping efficiency as compared to monocultures in GHG emission mitigation associated with vegetable production sector.

Assessing the impact of other leafy vegetables (lettuce and escarole) in Spain, which have a similar form of cultivation to that of the species evaluated in this study, Romero-Gámez et al. (2014) observed that fertilizers, mainly nitrogen fertilizers, were the main contributor sources to the GHG emissions associated with monoculture production of lettuce and escarole, as observed in the results obtained in the present study. In Greece, Foteinis and Chatzisymeon (2016) found that lettuce production, in conventional and organic systems, emitted about 1,893 and 1,603 kg CO_2_eq ha^-1^ cycle^-1^, respectively, with irrigation being the main contributor source with about 57.3 and 58.7% of GHG emissions, respectively. In the present study, irrigation was the third major contributor, accounting for 8 to 13% in all evaluated scenarios. This difference is mainly due to the electricity source used for the operation of the irrigation system. While in Greece electricity has high GHG emissions associated with manufacturing and consumption, due to its origin from fossil and non-renewable sources, in Brazil, most of the electricity (about 75%) comes from renewable sources (IPEA, 2019), resulting in lower GHG emissions associated with production and consumption during the use of the irrigation system, when compared to the production of other vegetables in Europe.

Concerning carbon footprint, Pereira et al. (2021) showed that, in Brazil, intercropping vegetables reduced the carbon footprint by up to 80% compared to monocultures. The results obtained in this study corroborate those found by Pereira et al. (2021) and confirm the intercropping as a more sustainable system for vegetable production than monocultures, when considering the ratio between yield and emissions per kilogram or kilocalorie of produced vegetables. The main challenge of modern agriculture is to reduce the environmental impacts generated by cropping systems, but without compromising crop yield. Thus, the results obtained in this study and the economic efficiency of intercropping demonstrated by Cecílio Filho et al. (2017) and Carlos et al. (2021) show that this cropping system meets environmental (climate changes) and economic aspects, which makes it an excellent alternative to the traditional monoculture production system of these vegetables.

In a literature review, Clune et al. (2017) reported that the carbon footprint to produce spinach varied from 0.51 to 0.54 kg CO_2_eq kg^-1^ spinach. Seo et al. (2017) verified that carbon footprint for organic spinach (*Spinacia oleracea*) production in Japan was 0.049 kg CO_2_eq 0.100 kg^-1^ spinach. The authors observed that fuel consumed in transportation contributed with 90% of the carbon footprint. It is necessary to highlight that this difference might be related to the boundaries established; while in the cited study the boundaries include fuel used in agricultural production phase and transportation of produce to the distribution center, in the present study the boundaries included the transportation over a distance of 1 kilometer inside the farm to the shed and, therefore, there is less fuel consumption.

When comparing the carbon footprint of Chinese kale (Brassicaceae) production in conventional and organic systems in Thailand, Yuttitham (2019) estimated values equal to 0.402±0.47 kg CO_2_eq kg^-1^ Chinese kale for conventional system and to 0.195±0.122 kg CO_2_eq kg^-1^ Chinese kale for organic system. As in the present study, the authors identified that in conventional system the main contributor sources were the use of fertilizers, fuel and irrigation. Nevertheless, the difference in the carbon footprint values when compared to those obtained in this study are related to higher GHG emissions due the fossil fuel used to generate the electricity consumed and the boundaries adopted, which also included the transportation to the distribution center.

As shown in our results, fertilizer use was the main responsible for the impact on GHG emissions associated with collard greens, spinach and chicory production, in open field, in the different evaluated scenarios. Reducing the use of synthetic fertilizers and increasing the efficiency of use of this input in the production of these vegetables may contribute to the mitigation of GHG emissions from this sector, especially in the state of São Paulo, Brazil. This state is the second major GHG emitter associated with the use of synthetic fertilizers (SEEG, 2020), with vegetable production being an important contributor; therefore, mitigation proposals for this sector should be more widely studied and implemented, such as use of organic fertilizers and N-fixing species, which generate less impact. For example, completely replacing N synthetic fertilizer with organic N fertilizers may reduce by 28% indirect GHG emissions associated with the manufacturing of N synthetic fertilizer in all evaluated scenarios. Additionally, crop rotation using N-fixing species such as *Crotalaria juncea*, *Cajanus cajan* and *Canavalia ensiformis* would take in about, respectively, 183.4, 143.6 and 169.4 kg N ha^-1^ in four years (equivalent to 45.8, 35.9 and 42.3 kg N ha^-1^ year^-1^), in dark red Oxisol (Silva et al., 2002), values that would represent reductions from 9 to 13% of the total synthetic N used in ICS and ICC scenarios (1 ha) and from 14 to 22% of the total in MCS and MCC scenarios (2 ha) (**Table 1**), varying according to N-fixing species used. Furthermore, it is important to highlight that in ICS and ICC scenarios the reduction in GHG emissions associated with the amount of fertilizers was about 25% compared to those of MCS and MCC scenarios, proving that intercropping of vegetables is also a promising technology for mitigating GHG emissions associated with synthetic fertilizers.

Fuel (diesel) consumption is also a great contributor to GHG emissions associated with vegetable production in the evaluated systems. A few alternatives aiming to reduce GHG emissions associated with this input would be to reduce the intensity and frequency of soil tillage with each new production cycle, starting to adopt reduced tillage practices, such as no-tillage, hence reducing diesel consumption. Looking ahead to future changes in the types of machines and engines used in agriculture, another mitigation alternative would be the use of hybrid tractors or tractors fully powered by fuels from non-fossil and more sustainable sources such as ethanol and renewable electricity (Hoy et al., 2014). Additionally, it is important to emphasize that production in intercropping system is also an efficient practice for mitigating GHG emissions associated with fuels, as in the results of this study, the reduction of GHG emissions associated with diesel, in intercropping scenarios, varied from 48 to 52% when compared to monoculture scenarios.

There are some limitations for a large-scale implementation of intercropping in vegetable production, as for vegetable production using this system to be economically viable, it is necessary to have temporal and spatial complementarity between the associated species. Thus, regional studies, such as those published by Cecílio Filho et al. (2017) and Carlos et al. (2021), should be carried out in order to define the proper management and synergy between species, since factors such as climate, competition for water, light and nutrients, especially temperature, may affect the speed of growth and development of the intercropped species and, consequently, influence yield.

A few factors such as the methodology (tiers) used in studies estimating GHG emission and carbon footprint, functional unit and boundaries adopted, may imply variations in calculating total GHG emitted or obtaining exact values of the impact on climate change/global warming (Bartzas et al., 2015; Notarnicola et al., 2017; Adewale et al., 2018) and, therefore, such limitations should be considered in the results obtained in this study. However, despite this issue, this methodology is considered the one that best suits this type of analysis, being widely accepted and used (Adewale et al., 2018).

The results presented in this study provide information about the contribution of these vegetables to GHG emissions from agriculture in Brazil and may help in future studies with broader projections of the impact of the vegetable production sector on Brazilian GHG emissions. In addition, this study demonstrates that the intercropping of collard greens, New Zealand spinach and chicory is an excellent alternative to monocultures of these vegetables, which may be part of the implementation strategies of more integrated and sustainable systems in Brazil and contribute to meet the global objectives for food safety of population and support the sustainable development of agriculture.

## Conclusions

The scenarios of vegetable cultivation in intercropping for collard greens, New Zealand spinach and chicory based on the parameters of this study accounted for 32% lower GHG emissions when compared to monoculture production scenarios for the same species of vegetables, during one year of cultivation in open field. The use of fertilizers, fuel (diesel), and electricity and materials used in irrigation are the main contributor sources to GHG emissions and carbon footprint, in all evaluated scenarios. The carbon footprint (in kg CO_2_eq kg^-1^ vegetables) in intercropping production scenarios of collard greens and spinach (ICS – 0.030 kg CO_2_eq kg^-1^ vegetables year^-1^) and collard greens and chicory (ICC – 0.033 kg CO_2_eq kg^-1^ of vegetables year^-1^) was 63 and 54% lower than in the scenarios of their respective monocultures (MCS – 0.082 kg CO_2_eq kg^-1^ of vegetables year^-1^ and MCC – 0.071 kg CO_2_eq kg^-1^ of vegetables year^-1^), respectively. Strategies aiming to reduce the impact of the production of these vegetables on GHG emissions should prioritize reducing the use of fertilizers, mainly nitrogen ones, through practices such as crop rotation with N-fixing species and greater use of organic fertilizers; reduce fuel consumption (diesel), by reducing soil tillage operations; and opt for more integrated cultivation systems such as intercropping, which promote lower GHG emissions compared to monocultures, in addition to being possible to obtain greater yield in these systems.

## Data availability statement

The original contributions presented in the study are included in the article/**Supplementary Material**. Further inquiries can be directed to the corresponding author.

## Author contributions

BP: investigation, data curation, writing-original draft. AC: conceptualization, methodology, writing-review and editing, supervision. NL: methodology, data curation, writing-review and editing, supervision. EF: methodology, data curation, writing-review and editing, validation. All authors contributed to the article and approved the submitted version.

## Funding

We would like to thank the Coordination for the Improvement of Higher Education Personnel (CAPES) for financial support.

## Conflict of interest

The authors declare that the research was conducted in the absence of any commercial or financial relationships that could be construed as a potential conflict of interest.

## Publisher’s note

All claims expressed in this article are solely those of the authors and do not necessarily represent those of their affiliated organizations, or those of the publisher, the editors and the reviewers. Any product that may be evaluated in this article, or claim that may be made by its manufacturer, is not guaranteed or endorsed by the publisher.

## References

[B1] Adewale C. Reganold J. P. Higgins S. Evans R. D. Carpenter-Boggs L. (2018). Improving carbon footprinting of agricultural systems: Boundaries, tiers, and organic farming. Environ. Impact Assess Rev. 71, 41–48. doi: 10.1016/j.eiar.2018.04.004

[B2] Bartzas G. Zaharaki D. Komnitsas K. (2015). Life cycle assessment of open field and greenhouse cultivation of lettuce and barley. Inf. Process Agric. 2, 191–207. doi: 10.1016/j.inpa.2015.10.001

[B3] Bisbis M. B. Gruda N. Blanke M. (2018). Potential impacts of climate change on vegetable production and product quality – a review. J. Clean Prod. 170, 1602–1620. doi: 10.1016/j.jclepro.2017.09.224

[B4] Carlos T. J. Cecílio Filho A. B. Passos D. dos R. C. Reis, I. dos S. (2021). Collard greens and chicory intercropping efficiency as a function of chicory (*Cichorium intybus*) transplant time. Rev. FCA UNCuyo 53, 91–99. doi: 10.48162/rev.39.043

[B5] Cecílio Filho A. B. Bianco M. S. Tardivo C. F. Pugina G. C. M. (2017). Agronomic viability of new Zealand spinach and kale intercropping. An. Acad. Bras. Cienc. 89, 2975–2986. doi: 10.1590/0001-3765201720160906 28876391

[B6] CEPAGRI (2006)Centro de pesquisa meteorológicas e climaticas aplicadas a agri- cultura. In: Clima dos municípios paulistas (Accessed November 16, 2020).

[B7] CETESB (2018) Companhia ambiental do estado de são paulo. Available at: https://cetesb.sp.gov.br/veicular/wp-content/uploads/sites/6/2013/12/Relatorio-Emissoes-Veiculares-2015-v4_.pdf (Accessed March 10, 2021).

[B8] Cheng K. Pan G. Smith P. Luo T. Li L. Zheng J.. (2011). Carbon footprint of china’s crop production-an estimation using agro-statistics data over 1993-2007. Agric. Ecosyst. Environ. 142, 231–237. doi: 10.1016/j.agee.2011.05.012

[B9] Clavreul J. Butnar I. Rubio V. King H. (2017). Intra- and inter-year variability of agricultural carbon footprints – a case study on field-grown tomatoes. J. Clean Prod. 158, 156–164. doi: 10.1016/j.jclepro.2017.05.004

[B10] Clune S. Crossin E. Verghese K. (2017). Systematic review of greenhouse gas emissions for different fresh food categories. J. Clean Prod. 140, 766–783. doi: 10.1016/j.jclepro.2016.04.082

[B11] Do Carmo H. F. Madari B. E. Wander A. E. Moreira F. R. B. de O. A. C. da P. M.. (2016). Balanço energético e pegada de carbono nos sistemas de produção integrada e convencional de feijão-comum irrigado. Pesqui. Agropecuária Bras. 51, 1069–1077. doi: 10.1590/s0100-204x2016000900006

[B12] FAO (2019) Faostat crops database. Available at: http://www.fao.org/faostat/en/#data/QC (Accessed April 25, 2021).

[B13] Foteinis S. Chatzisymeon E. (2016). Life cycle assessment of organic versus conventional agriculture. a case study of lettuce cultivation in Greece. J. Clean Prod. 112, 2462–2471. doi: 10.1016/j.jclepro.2015.09.075

[B14] Hoy R. Rohrer R. Liska A. Luck J. Isom L. Keshwani D. (2014). Agricultural industry advanced vehicle technology: Benchmark study for reduction in petroleum use (Idaho Falls, USA: Idaho National Laboratory, University of Nebraska).

[B15] IBGE (2017) Instituto brasileiro de geografia e estatística. Available at: https://sidra.ibge.gov.br/home/pimpfbr/brasil. (Accessed April 25, 2021).

[B16] IPCC (2006). “Guidelines for national greenhouse gas inventories,” in IPCC national greenhouse gas inventories programme. Eds. Eggleston S. Buendia L. Miwa K. Ngara T. Tanabe K. (Hayama, Japan: Institute for Global Environmental Strategies (IGES), 664.

[B17] IPCC Myhre G. Shindell D. Bréon F. M. Collins W. Fuglestvedt and J.. (2013). “Anthropogenic and natural radiative forcing,” in Climate change 2013: the physical science basis. contribution of working group I to the fifth assessment report of the intergovernmental panel on climate change. Eds. Stocker T. F. Qin D. Plattner G. K. Tignor M. Allen S. K. Boschung J. Nauels A. Xia Y. Bex V. Midgley P. M. (United Kingdom and New York, NY, USA: Cambridge University Press, Cambridge).

[B18] IPEA (2019)Instituto de pesquisa econômica aplicada. In: ODS 7 - assegurar o acesso confiáavel, sustentável, moderno e a preço acessível à energia para todos. O que mostra o retrato do brasil? Available at: http://www.ipea.gov.br/portal/ (Accessed July 17, 2021).

[B19] Jeswani H. K. Espinoza-Orias N. Croker T. Azapagic A. (2018). Life cycle greenhouse gas emissions from integrated organic farming: A systems approach considering rotation cycles. Sustain. Prod. Consum. 13, 60–79. doi: 10.1016/j.spc.2017.12.003

[B20] Lessa A. C. R. Madari B. E. Paredes D. S. Boddey R. M. Urquiaga S. Jantalia C. P.. (2014). Bovine urine and dung deposited on Brazilian savannah pastures contribute differently to direct and indirect soil nitrous oxide emissions. Agric. Ecosyst. Environ. 190, 104–111. doi: 10.1016/j.agee.2014.01.010

[B21] Lo-Iacono-Ferreira V. G. Viñoles-Cebolla R. Bastante-Ceca M. J. Capuz-Rizo S. F. (2020). Transport of Spanish fruit and vegetables in cardboard boxes: A carbon footprint analysis. J. Clean Prod. 244, 118784. doi: 10.1016/j.jclepro.2019.118784

[B22] Macedo I. C. Seabra J. E. A. Silva J. E. A. R. (2008). Green house gases emissions in the production and use of ethanol from sugarcane in Brazil: The 2005/2006 averages and a prediction for 2020. Biomass Bioenergy 32, 582–595. doi: 10.1016/j.biombioe.2007.12.006

[B23] Martin-Gorriz B. Gallego-Elvira B. Martínez-Alvarez V. Maestre-valero J (2020). Life cycle assessment of fruit and vegetable production in the region of murcia (south-east Spain) and evaluation of impact mitigation practices F. J. Clean Prod. 265, 1–14. doi: 10.1016/j.jclepro.2020.121656

[B24] MCTI (2020) Ministério da ciência, tecnologia e inovação (Accessed January 28, 2021).

[B25] Nascimento C. S. Cecílio Filho A. B. Mendoza-Cortez J. W. Nascimento C. S. Bezerra Neto F. Grangeiro L. C. (2018). Effect of population density of lettuce intercropped with rocket on productivity and land-use efficiency. PloS One 13, 1–14. doi: 10.1371/journal.pone.0194756 PMC5919433 29698401

[B26] Notarnicola B. Sala S. Anton A. McLaren S. J. Saouter E. Sonesson U. (2017). The role of life cycle assessment in supporting sustainable agri-food systems: A review of the challenges. J. Clean Prod. 140, 399–409. doi: 10.1016/j.jclepro.2016.06.071

[B27] Ntinas G. K. Neumair M. Tsadilas C. D. Meyer J. (2017). Carbon footprint and cumulative energy demand of greenhouse and open-field tomato cultivation systems under southern and central European climatic conditions. J. Clean Prod. 142, 3617–3626. doi: 10.1016/j.jclepro.2016.10.106

[B28] Pereira B. D. J. Cecílio Filho A. B. La Scala N. (2021). Greenhouse gas emissions and carbon footprint of cucumber, tomato and lettuce production using two cropping systems. J. Clean Prod. 282, 124517. doi: 10.1016/j.jclepro.2020.124517

[B29] Pishgar-Komleh S. H. Akram A. Keyhani A. Raei M. Elshout P. M. F. Huijbregts M. A. J.. (2017). Variability in the carbon footprint of open-field tomato production in Iran - a case study of alborz and East-Azerbaijan provinces. J. Clean Prod. 142, 1510–1517. doi: 10.1016/j.jclepro.2016.11.154

[B30] Posen I. D. Jaramillo P. Griffin W. M. (2017). Greenhouse gas mitigation for U.S. plastics production: Energy first, feedstocks later. Environ. Res. Lett. 12, 1–12. doi: 10.1088/1748-9326/aa60a7 36204013

[B31] Romero-Gámez M. Audsley E. Suárez-Rey E. M. (2014). Life cycle assessment of cultivating lettuce and escarole in Spain. J. Clean Prod. 73, 193–203. doi: 10.1016/j.jclepro.2013.10.053

[B32] SEEG (2020)Sistema de estimativas de emissões de gases de efeito estufa. In: Análises das emissões brasileiras de gases de efeito estufa e suas implicações para as metas do brasil 1970-2019. Available at: http://seeg.eco.br/ (Accessed March 28, 2021).

[B33] Seo Y. Ide K. Kitahata N. Kuchitsu K. Dowaki K. (2017). Environmental impact and nutritional improvement of elevated CO2 treatment: A case study of spinach production. Sustainability 9, 1–9. doi: 10.3390/su9101854

[B34] Seo Y. Someya Y. Dowaki K. (2019). Environmental impacts and consumer preference for sustainably cultivated Japanese mustard spinach, komatsuna. J. Environ. Manage. 231, 364–369. doi: 10.1016/j.jenvman.2018.10.077 30368145

[B35] Silva J. A. A. Vitti G. C. Stuchi E. S. Sempionato O. R. (2002). Reciclying and incorporation of nutrients to the soil in orchard of ‘Pera’ orange by cultivation with cover crops. Rev. Bras. Frutic. 24, 225–230. doi: 10.1590/S0100-29452002000100048

[B36] TACO (2011)Tabela brasileira de composição de alimentos. In: NEPA - UNICAMP. Available at: https://www.nepa.unicamp.br/taco/tabela.php?ativo=tabela (Accessed March 23, 2021).

[B37] Tasca A. L. Nessi S. Rigamonti L. (2017). Environmental sustainability of agri-food supply chains: An LCA comparison between two alternative forms of production and distribution of endive in northern Italy. J. Clean Prod. 140, 725–741. doi: 10.1016/j.jclepro.2016.06.170

[B38] Trani P. E. Van Raij B. Cantarella H. De Figueiredo G. J. B. (2018). Hortaliças, recomendação de calagem a adubação para o estado de são paulo (Instituto Agronômico Campinas (IAC), Campinas: CATI) 88.

[B39] UN (2015) United nations. sustainable development goals. Available at: https://www.un.org/sustainabledevelopment/ (Accessed June 10, 2021).

[B40] Vico A. Sáez J. A. Pérez-Murcia M. D. Martinez-Tomé J. Andreu-Rodríguez J. Agulló E.. (2020). Production of spinach in intensive Mediterranean horticultural systems can be sustained by organic-based fertilizers without yield penalties and with low environmental impacts. Agric. Syst. 178, 102765. doi: 10.1016/j.agsy.2019.102765

[B41] Yuttitham M. (2019). Comparison of carbon footprint of organic and conventional farming of chinese kale. Environ. Nat. Resour. J. 17, 78–92. doi: 10.32526/ennrj.17.1.2019.08

[B42] Zarei M. J. Kazemi N. Marzban A. (2019). Life cycle environmental impacts of cucumber and tomato production in open-field and greenhouse. J. Saudi Soc Agric. Sci. 18, 249–255. doi: 10.1016/j.jssas.2017.07.001

