# Supplementary material for: Greenhouse gas emissions and carbon footprint of collard greens, spinach and chicory production systems in Southeast of Brazil
Source: Front Plant Sci. 2022 Nov 2;13:1015307. doi: 10.3389/fpls.2022.1015307 (PMC9667872; doi:10.3389/fpls.2022.1015307)
Supplement: Supplementary file 1 [file DataSheet_1.docx]

**SUPPLEMENTARY MATERIAL**

**Supplementary material – Table 1 |** Direct and indirect emission sources and factors considered in the calculation of GHG emissions from collard greens, New Zealand spinach and chicory production in intercropping and monoculture scenarios.

| **Source** | **Unit** | **Emission factor** | **Reference** |
| --- | --- | --- | --- |
| **Direct emission** |  |  |  |
| N fertiliser | kg N_2_O-N kg^-1^ | N applied – 0.01  N volatilized – 0.001  N leached – 0.00225 | IPCC (2006) |
| Manure | kg N_2_O-N kg^-1^ | 0.001 | Lessa et al. (2014) |
| Limestone | kg CO_2_ kg^-1^ | 0.13 | IPCC^a^ (2006) |
| Diesel | kg CO_2_ L^-1^ | 2.603 | CETESB^b^ (2018) |
|  |  |  |  |
| **Indirect emission** |  |  |  |
| N fertiliser | kg CO_2_eq kg^-1^ N | 3.97 | Macedo et al. (2008) |
| P fertiliser | kg CO_2_eq kg^-1^ P_2_O_5_ | 1.13 | Macedo et al. (2008) |
| K fertiliser | kg CO_2_eq kg^-1^ K_2_O | 0.71 | Macedo et al. (2008) |
| Limestone | kg CO_2_eq kg^-1^ | 0.01 | Macedo et al. (2008) |
| Fungicide | kg CO_2_eq kg^-1^ i.a^d^ | 28.29 | Do Carmo et al. (2016) |
| Insecticide | kg CO_2_eq kg^-1^ i.a^d^ | 29.00 | Macedo et al. (2008) |
| Diesel | kg CO_2_ L^-1^ | 0.581 | Macedo et al. (2008) |
| Electricity-Irrigation | t CO_2_ MWh^-1^ | 0.0617 | MCTI^c^ (2020) |
| Irrigation pipes | kg CO_2_eq kg^-1^ PVC^e^ | 2.20 | Posen et al. (2017) |
| Irrigation sprinkler | kg CO_2_eq kg^-1^ LDPE^f^ | 1.80 | Posen et al. (2017) |
| Seedling trays | kg CO_2_eq kg^-1^ LDPE^f^ | 1.80 | Posen et al. (2017) |
| Iron | CO_2_eq kg^-1^ iron | 1.35 | IPCC (2006) |
| Film plastic | kg CO_2_eq kg^-1^ plastic | 5.18 | Cheng et al. (2011) |

^a^ Intergovernmental Panel on Climate Change.

^b^ São Paulo State Environmental Company.

^c^ Ministry of Science, Technology and Innovations.

^d^ Active ingredient.

^e^ Polyvinyl Chloride.

^f^ Low-density polyethylene.

**Supplementary material – Table 2 |** Fuel consumption (diesel) in agricultural operations using a MF 275 tractor (77 cv) in the production of collard greens (1 cycle per year), New Zealand spinach (2 cycles per year) and chicory (3 cycles per year) in intercropping and monoculture scenarios.

| **Operations** | **Operations time** (hours) | **Diesel consumption**  (L h^-1^) | **ICS**  (L ha^-1^ year^-1^) | **MCS**  (L ha^-1^ year^-1^) | **ICC**  (L ha^-1^ year^-1^) | **MCC**  (L ha^-1^ year^-1^) |
| --- | --- | --- | --- | --- | --- | --- |
| Ploughing | 3 | 11.0 | 33.0 | 99.0 | 33.0 | 132.0 |
| Harrowing | 2 | 11.0 | 22.0 | 66.0 | 22.0 | 88.0 |
| Bedding | 1 | 11.0 | 33.0 | 99.0 | 33.0 | 132.0 |
| Limestone application | 1 | 11.0 | 11.0 | 22.0 | 11.0 | 22.0 |
| Manure application | 1 | 11.0 | 11.0 | 22.0 | 11.0 | 22.0 |
| Harvest transportation | 3 | 11.0 | 66.0 | 66.0 | 99.0 | 99.0 |
| Seedlings transportation | - | 4 km L^-1^ | 25.0^a^ | 25.0^a^ | 37.5^b^ | 37.5^b^ |
| Fertilisers transportation | - | 4 km L^-1^ | 12.5 | 12.5 | 12.5 | 12.5 |
| **Total** |  |  | **213.5** | **411.5** | **259.0** | **545.5** |

^a^ Applied two times per year.

^b^ Applied three times per year.
